# Impact of Enriched Environment on Murine T Cell Differentiation and Gene Expression Profile

**DOI:** 10.3389/fimmu.2016.00381

**Published:** 2016-09-30

**Authors:** Lorenza Rattazzi, Giuseppa Piras, Samuel Brod, Koval Smith, Masahiro Ono, Fulvio D’Acquisto

**Affiliations:** ^1^William Harvey Research Institute, Barts and the London School of Medicine and Dentistry, Queen Mary University of London, London, UK; ^2^Department of Life Science, Faculty of Natural Science, Imperial College of Science, Technology and Medicine, London, UK

**Keywords:** CD4 T cells, enriched environment, microarray, cytokines, resolution of inflammation

## Abstract

T cells are known to be plastic and to change their phenotype according to the cellular and biochemical milieu they are embedded in. In this study, we transposed this concept at a macroscopic level assessing whether changes in the environmental housing conditions of C57/BL6 mice would influence the phenotype and function of T cells. Our study shows that exposure to 2 weeks in an enriched environment (EE) does not impact the T cell repertoire *in vivo* and causes no changes in the early TCR-driven activation events of these cells. Surprisingly, however, T cells from enriched mice showed a unique T helper effector cell phenotype upon differentiation *in vitro*. This was featured by a significant reduction in their ability to produce IFN-γ and by an increased release of IL-10 and IL-17. Microarray analysis of these cells also revealed a unique gene fingerprint with key signaling pathways involved in autoimmunity being modulated. Together, our results provide first evidence for a specific effect of EE on T cell differentiation and its associated changes in gene expression profile. In addition, our study sheds new light on the possible mechanisms by which changes in environmental factors can significantly influence the immune response of the host and favor the resolution of the inflammatory response.

## Introduction

Over the past decade, a growing body of research has identified that many immune cells are able to dynamically alter their form and function in response to changes in the local microenvironment. Such functional and phenotypic plasticity has been observed in cells of both the innate (1–3) and adaptive (4–7) immune system. In the context of the adaptive immune response, an ever-growing number of studies have investigated the experimental conditions (such as skewing cytokines, antigen-presenting cells, or receptor co-stimulation) that favor the differentiation of T cells into a specific effector T helper (Th) type. One such example is the multitude of *in vitro* and *in vivo* studies on Th17 cells, a differentiated and apparently fully committed Th cell phenotype, which has been shown to switch between a pathogenic, inflammatory and protective, regulatory functions (and *vice versa*) in accordance with the conditions in which they are cultured (8–11).

While the concept of immune cell mutability in response to the specific tissue and cellular surrounding has been widely explored *in vitro* (12, 13), little is known about the influence of the external “*de-corpore*” (trans. Out of the body) environmental factors. This is surprising considering that a great deal of literature suggests that after genetics, factors such as pollution (14–16), geographical location (17, 18), psychological state (19–22), and social status (23–26) are each key determinants in the etiology of autoimmune disorders.

Aiming to understand how these environmental factors modulate the immune response, we decided to investigate the effect of a well-established experimental paradigm – the enriched environment (EE) (27, 28) – on T cell function. Initially described by Donald Hebb [for an extensive historical and scientific review on the topic, see Ref. (29)], EE uses one or more altered housing conditions to provide an “enriched” sensory, physical, and/or social environment for an animal. Such enrichment materials can include tunnels, wheels, ladders, and further enclosed apparatus that together provide a richer, “multisensorial” standard of living compared to a classical lab enclosure.

Studies on the biological effects of environmental enrichment have identified a number of beneficial effects on a variety of physiological parameters, including neuronal growth and development, vascular inflammation, and metabolic syndromes (27, 28, 30). Strikingly, in the majority of these studies, such beneficial effects are demonstrated by an improved capability to recover from experimental pathologies, such as trauma (31), cellular degeneration (32–40), and neoplasms (41, 42). This suggests an improved capacity for healing to be a common denominator for the potential therapeutic effects of an EE. Interestingly, several studies have shown that immune activation is often paramount to an efficient healing process (43, 44).

Our results show that a period as short as 2 weeks of housing in an EE causes significant changes to the gene expression profile of T cells as well as their effector function, shifting their profiles from a classical Th1 phenotype toward a more intermediate state featured by a reduction in IFN-γ and an increase in IL-10 and IL-17 production. These results provide the first evidence of epigenetic changes induced by EE in T cells while also providing clues about their possible contribution to a faster resolution of immune and inflammatory diseases.

## Materials and Methods

### Mice

For all the experiments, we used 6-week-old C57BL/6 male mice purchased from Charles River. Mice were housed in groups of maximum six animals per cage under specific pathogen-free conditions and with free access to food and water. Mice were housed for at least 7 days prior to testing. All tests were conducted in a blinded fashion and according to the UK Animals Scientific Procedures Act, 1986. The local biological service unit at Queen Mary University of London approved all experimental protocols.

### Enriched Environment Normal Environment

Mice (five per cage) were housed either in normal environment (NE) or EE for 2 weeks according to Sztainberg and Chen (45) with some modifications. Briefly, NE consisted of a standard mouse cage (W × D × H – 40 cm × 16.5 cm × 17.4 cm) filled with 2–3 cm of sawdust and nesting material (paper strips); EE consisted of a wider cage (W × D × H – 40 cm × 25 cm × 20 cm) containing wood shavings in addition to sawdust and different nesting materials and toys. More specifically, one colored transparent plastic nest-box (Superpet Puzzle Playground; Superpet; Amazon UK), one fabric tube (Small Crinkle Activity Tunnel, Amazon UK), one running wheel (Kaytee/Superpet Silent Spinner, 4.5-inch, Mini; Amazon, UK), and one wood hamster swing (Trixie Hamster Wood Seesaw Toy, Amazon, UK). The toys were changed after 1 week and replaced with new ones. The weight of the mice was recorded every other day for 2 weeks.

### Behavioral Tests: Open Field and Marble-Burying Tests

**Figure 1:**
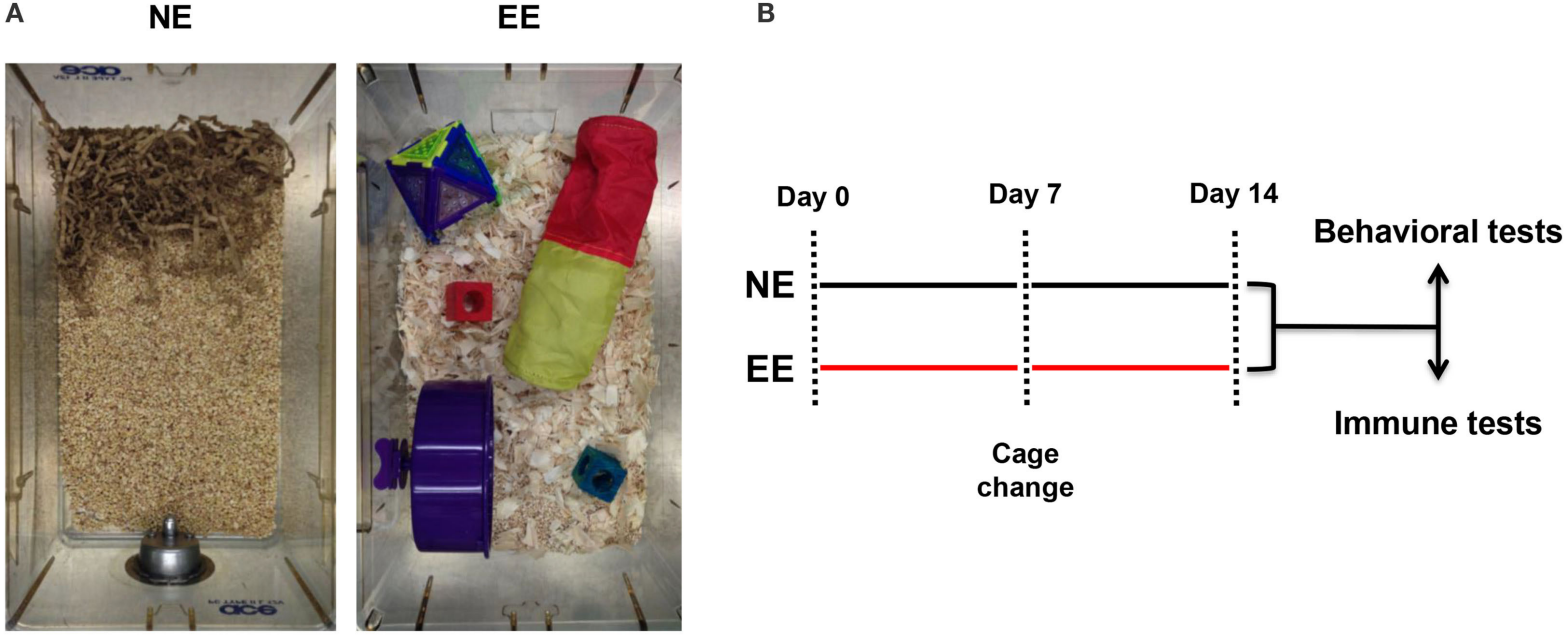
**Normal environment and enriched environment**. Pictures of the housing conditions of control (NE) or enriched (EE) mice **(A)** and schematic representation of the experimental paradigm used for this study including the two main readouts used at the end of 2 weeks **(B)**.

If not otherwise stated, tests were performed double-blind every other day during the light phase of the light–dark cycle, as previously described and recommended (46). All the efforts were made to minimize mouse discomfort in these behavioral experiments. Mice were brought to the testing room at least 30 min before the start of the test session to allow habituation to the testing environment. Unless otherwise specified, standard lighting (about 50 lux) and quiet conditions were maintained throughout each experiment. The open field test (OFT) is an ethologically based paradigm that provides objective measures of exploratory behavior as well as a valid initial screen for anxiety-related behavior in rodents and was carried out as previously described. The apparatus consisted of a white PVC arena (50 cm × 30 cm × 20 cm) divided into 10 cm × 10 cm squares (*n* = 15). The three central squares defined the “center” region. Each mouse was placed in a corner square, facing the wall, and observed and recorded for 3 min. The total number of squares crossed (all four paws in), total number of rears (defined as both front paws off the ground, but not as a part of grooming), and number of center crossings was recorded. The walls and floor of the arena were thoroughly cleaned between each trial. The marble-burying test (MBT) is thought to reflect repetitive and perseverative behavior, possibly related to compulsions and/or anxiety disorders (47). The test was carried out as described by Deacon and colleagues (48) with some modifications. Briefly, mice were individually placed in a clear plastic box (14 cm × 10 cm × 11 cm) filled with ~5-cm depth of wood chip bedding lightly pressed to give a flat surface (see Figure 1). Fifteen 1.5-cm diameter glass marbles were placed on the surface, evenly spaced, each about 4 cm apart, so to form 5 rows of 3. The latency to start digging (defined as the mouse digging the bedding with front and hind paws for more than 1 s), the total number of digging bouts, and the number of buried marbles (to two-third of their depth) were manually recorded during the 10-min test.

### Flow Cytometric Analysis

Lymphocytes from thymus, lymph nodes, or spleen were stained in 100 μl of FACS buffer (PBS containing 5% FCS and 0.02% of NaN_2_). The antibodies used were anti-CD3 PE (clone 145-2C11, eBioscience), anti-CD4 FITC (clone GK 1.5, eBioscience), anti-CD8 Cy5 (clone 53-6.7, eBioscience), anti-CD25 FITC (clone PC61, BioLegend), and anti-CD69 PE (clone H1.2F3, eBioscience). Cells were labeled with the appropriate concentration of conjugated antibodies for 1 h at 4°C as previously described (49). After labeling, cells were washed and analyzed. Cells were first gated for singlets (FSC-H vs. FSC-A) and lymphocytes (SSC-A vs. FSC-A). The lymphocyte gate was further analyzed for their expression of CD3, CD4, or CD8. In all the experiments, stained cells were acquired with FACScalibur flow cytometer and analyzed using FlowJo™ software (Tree Star, Inc., Oregon Corporation).

### Cell Treatment and Cytokine Measurement

Freshly isolated lymphocytes were seeded in the same number with or w/o plate-bound anti-CD3/28 antibodies in the concentration of 0.5 μg/ml. After an overnight incubation at 37°C, cells were stained for FACS analysis, supernatants were collected, and cytokine levels were measured. For the skewing experiments, cells were cultured in Th1 conditions as previously described (50). Briefly, cells were stimulated with plate-bound anti-CD3/28 antibodies in the concentration of 1 μg/ml in the presence of IL-2 (10 ng/ml), IL-12 (3.4 ng/ml), and anti-IL-4 (2 μg/ml). In some experiments, cells were skewed in Th2 [IL-4 (10 ng/ml); IL-2 (10 ng/ml); anti-INFγ (2 μg/ml)] or Th17 [IL-6 (10 ng/ml); anti-IL-4 (2 μg/ml); anti-INFγ (2 μg/ml); TGF-β (5 ng/ml)] as previously described (51). After 72-h incubation at 37°C, supernatants were collected, and cytokine levels were measured. Cytokine levels were measured on undiluted supernatant using the Mouse Th1/Th2/Th17/Th22 16plex Kit Flowcytomix and according to the manufacturer’s instructions (eBioscience). For the intracellular staining, differentiated cells were stimulated with concanavalin-A (ConA, 5 μg/ml; Sigma-Aldrich, Dorset, England) in the presence of protein transport inhibitor brefeldin-A (1:1000; eBioscience) for 4 h. Con-A1 stimulated cells were pelleted, stained for CD4 (1:500) for 30 min, and fixed with 1% PFA for 10 min. Finally, cells were permeabilized and stained for 30 min in permeabilization buffer (composed by 0.1% saponin and 0.09% NaN_2_ in PBS, eBioscience, Hatfield, UK) containing conjugated antibodies (dil: 1:250) against IFNγ (clone XMG1.2, eBioscience) and IL-17 (eBioTC11-18H10.1, eBioscience). Labeled cells were washed, resuspended in FACS buffer containing 1% PFA, and analyzed by FACScalibur. Acquired data were analyzed by FlowJo™ software (Tree Star, Inc., Oregon Corporation).

### Corticosterone Measurement

Corticosterone concentrations were measured in diluted (1:32) plasma by EIA assay following the manufacturer’s instructions (Enzo Life Sciences).

### Microarray Analysis and Bioinformatics

Total RNA was extracted using RNeasy^®^ Microarray Tissue Mini Kit (Qiagen^®^, West Sussex, UK), and the labeled DNA was hybridized to Affymetrix MoGene1_0-st Gene Arrays (transcript version). Computational analysis was performed using Mac OS 10.6.8, and R version 3.1.0. affymetrix platform MoGene_1.0st transcript cluster Microarray data were normalized by rma of the Bioconductor package, affy. Differentially expressed genes (DEG) were identified by the Bioconductor package limma. Heatmap was generated by the function heatmap.2 of the CRAN package, gplots, using the complete-linkage clustering using the Euclidean distance. Pathway analysis was performed by a signaling pathway impact analysis, using the Bioconductor package, SPIA. Briefly, SPIA tests both the enrichment of genes in each pathway and the impact of the DEG on the signaling cascade, considering its topology (network) using a bootstrapping procedure of genes (*B* = 2000). These two tests generate two probability values, which are combined to generate a global probability, PG, and PG < 0.05 was considered significant. In other words, pathways with a significant probability value by SPIA are considered significantly perturbed (52).

#### RT-PCR

Total RNA was extracted according to the manufacturer’s protocol and reverse transcribed using 2-μg oligo(dT)15 primer, 10 units AMV reverse transcriptase, 40 units RNase inhibitor (all from Promega, Southampton, UK), and 1.25 mM each dNTP (Bioline, London, UK) for 45 min at 42°C. Real-time PCR was carried out using TaqMan Universal PCR master mix and fluorescent primers obtained from Quiagen (QuantiTect primers). Cycling conditions were set according to manufacturer’s instructions. Sequence-specific fluorescent signal was detected by an ABI Prism 7700Sequence Detector System. mRNA data were normalized relative to GADPH and then used to calculate expression levels. We used the comparative Ct method (53) to measure the gene transcription in samples. The results are expressed as relative units based on calculation of 2 − ΔΔCt, which gives the relative amount of gene normalized to endogenous control (GADPH) and to the sample with the lowest expression set as one.

### Data Analysis

Pairwise comparisons were made by *t*-test, while comparisons of more than two groups were analyzed using one-way ANOVA. Statistical significance was set at *p* ≤ 0.05, and all data are presented as mean ± SEM.

## Results

### Environmental Enrichment Does Not Cause Gross Changes to the T Cell Repertoire

Previous studies employing an EE have used a wide range of exposure times varying from hours to weeks (54–58). In this study, we used a 2-week EE protocol based on preliminary studies aimed at assessing the minimum time needed to see changes in T cell differentiation (data not shown) and based on previous investigations showing stable changes in the gene expression profile of the brain at this time point (58). At the end of the 2 weeks of enrichment or standard housing, mice were either subjected to behavioral and biochemical tests or assessed for their immune repertoire (see scheme in Figure 1).

**Figure 2:**
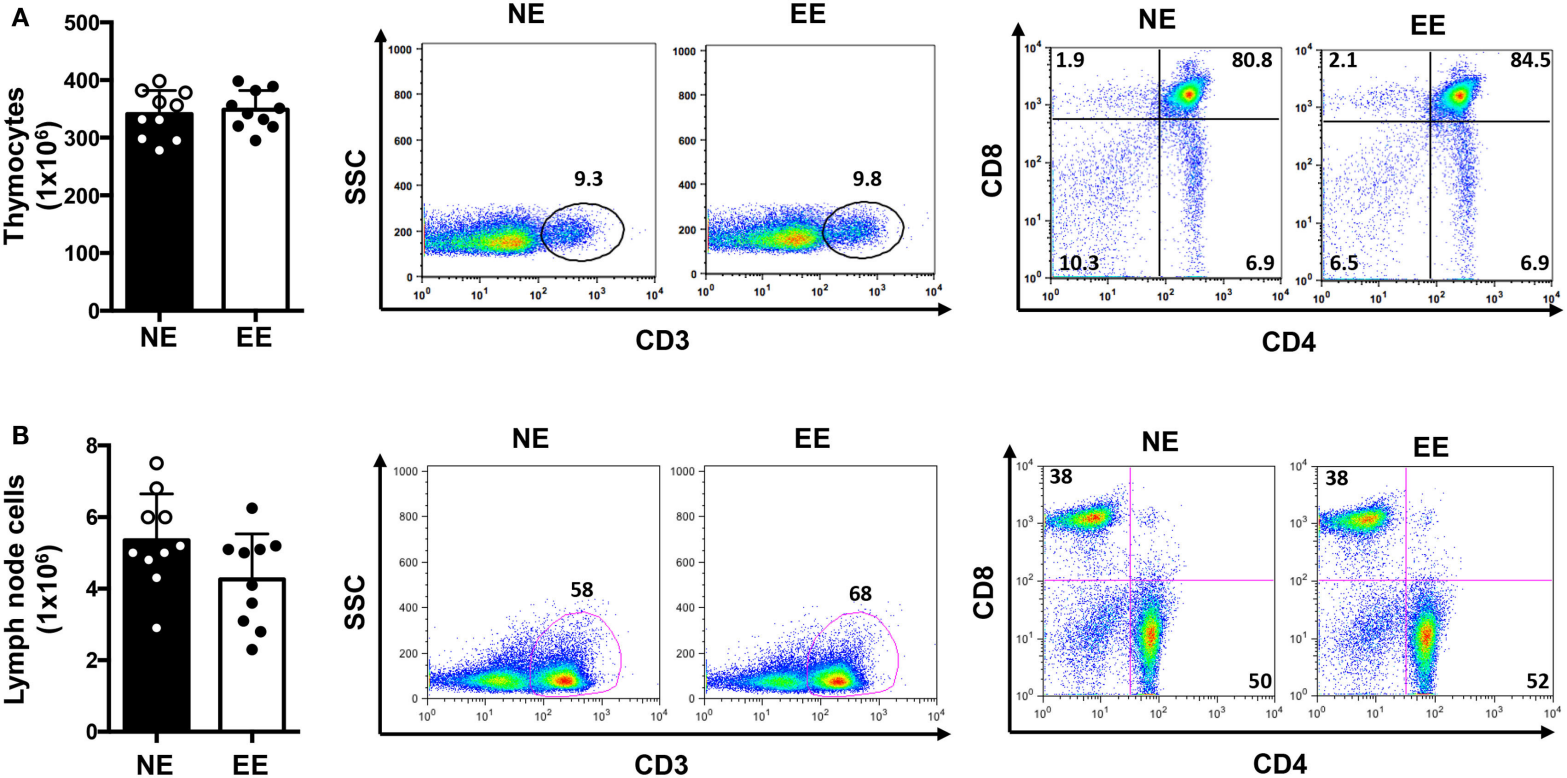
**T cell immune repertoire of C57/Bl6 mice after 2 weeks of EE**. The bar graphs on the left side show the absolute numbers of thymocytes **(A)** and lymph node **(B)** cells in NE and EE mice. The dot plots show the percentage of CD3+ T cells (middle panels) or the percentages of CD4+ and CD8+ T cells (right panels) collected from thymus (top panels) or lymph nodes (bottom panels) of NE and EE mice. The numbers in the plots show the percentages of gated populations. Data shown are from a single mouse and representative of *n* = 4 separate experiments with five mice per each group.

We first tested the possible effects of EE on the overall T cell repertoire *in vivo* looking at the CD3, CD4, or CD8 profile in the thymus and lymph nodes. There were no significant differences in the total cell number of either thymocytes or lymph node cells (Figures 2A,B, left panels, respectively). Flow cytometric analysis showed a small but non-significant increase (~5–10%) in relative percentages and number of CD3+ cells from EE compared to NE mice in both thymus and lymph nodes (Figures 2A,B, middle panels, respectively). Similarly, no differences in the ratio of CD4+ vs. CD8+ cell were found between groups (Figures 2A,B, right panels, respectively).

**Figure 3:**
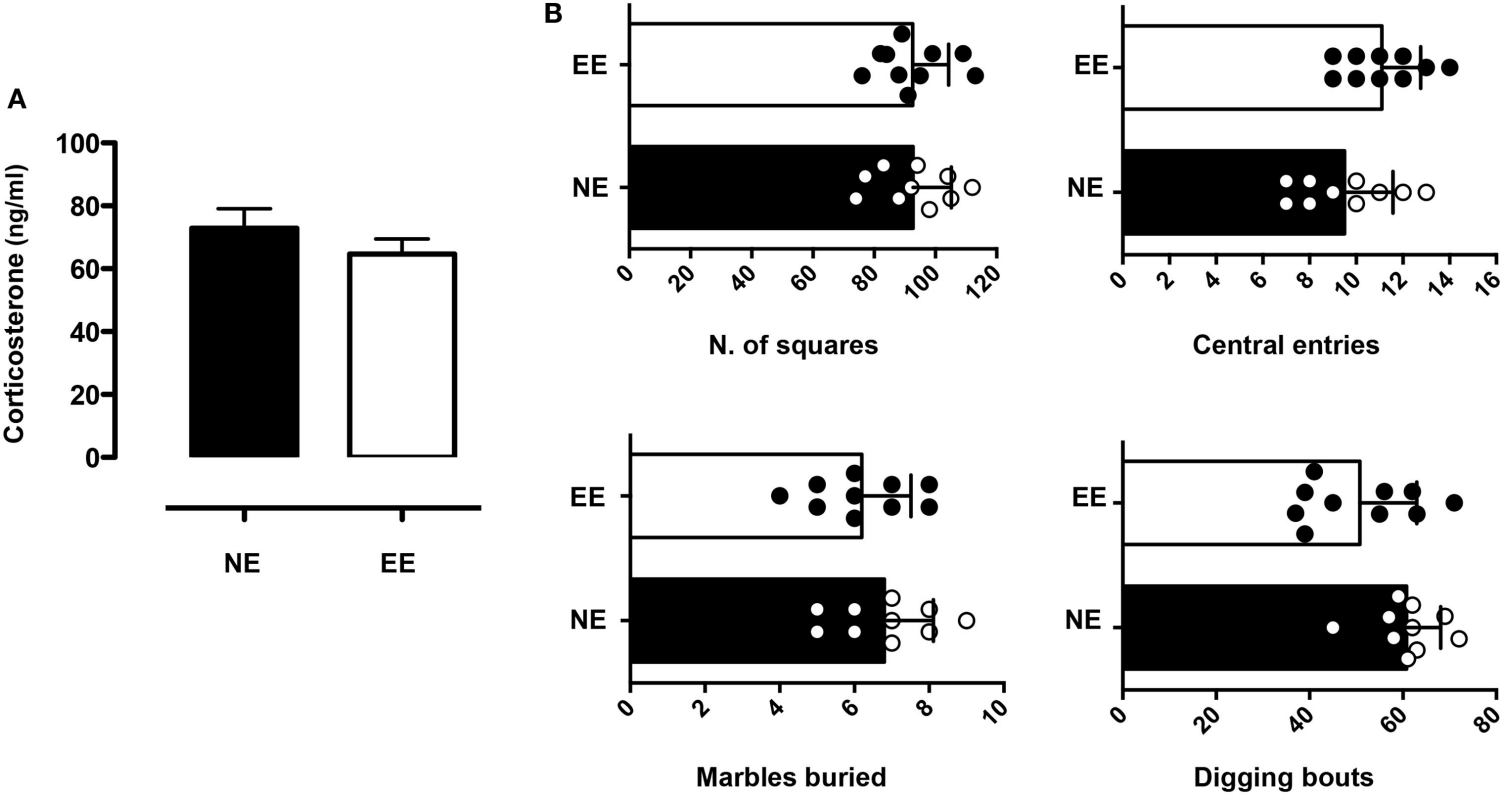
**Anxiety-like behavior and plasma corticosterone levels in C57/Bl6 mice after 2 weeks of EE**. **(A)** Levels of plasma corticosterone in mice at the end of 2 weeks housing in NE or EE conditions. Data shown are means ± SEM from *n* = 5 mice and representative of *n* = 3 separate experiments with similar results. **(B)** Mice were tested for the anxiety-like behavior at the end of their 2 weeks of NE or EE housing using the open field (top bar charts) and the light/dark shuttle box (bottom bar charts). The top-graphs show the number of squares traveled (left) or the number of central entries (right) in the open field after 5 min of testing. The bottom graphs show the number of light/dark transitions (left) and the time in the light (right) in the light and dark shuttle box at the end of 10-min test. Data shown means ± SEM from *n* = 10 mice and are representative of *n* = 3 separate experiments with similar results.

We continued this overall assessment of the effect of 2 weeks of EE by measuring its influence on anxiety-like behavior. Analysis of plasma corticosterone revealed levels of the hormone to be slightly lower (non-significant) in the EE group compared to NE (Figure 3A). Consistent with these results, we found no significant differences in anxiety-like behavior as evaluated by two separate assessors: the open field and MBT. Indeed, the number of central entries [directly proportional to a reduced state of anxiety-like behavior (59)] and the overall horizontal activity (evaluated by the number of squares crossed) of NE and EE mice in the OFT were comparable (Figure 3B, top left and right panel, respectively). Similarly, in the MBT, the number of buried marble or digging bouts (both parameters of increased anxiety-like behavior) showed no significant differences (Figure 3B, bottom left and right panel, respectively).

### Effect of Environmental Enrichment on the Early T Cell Activation Events

**Figure 4:**
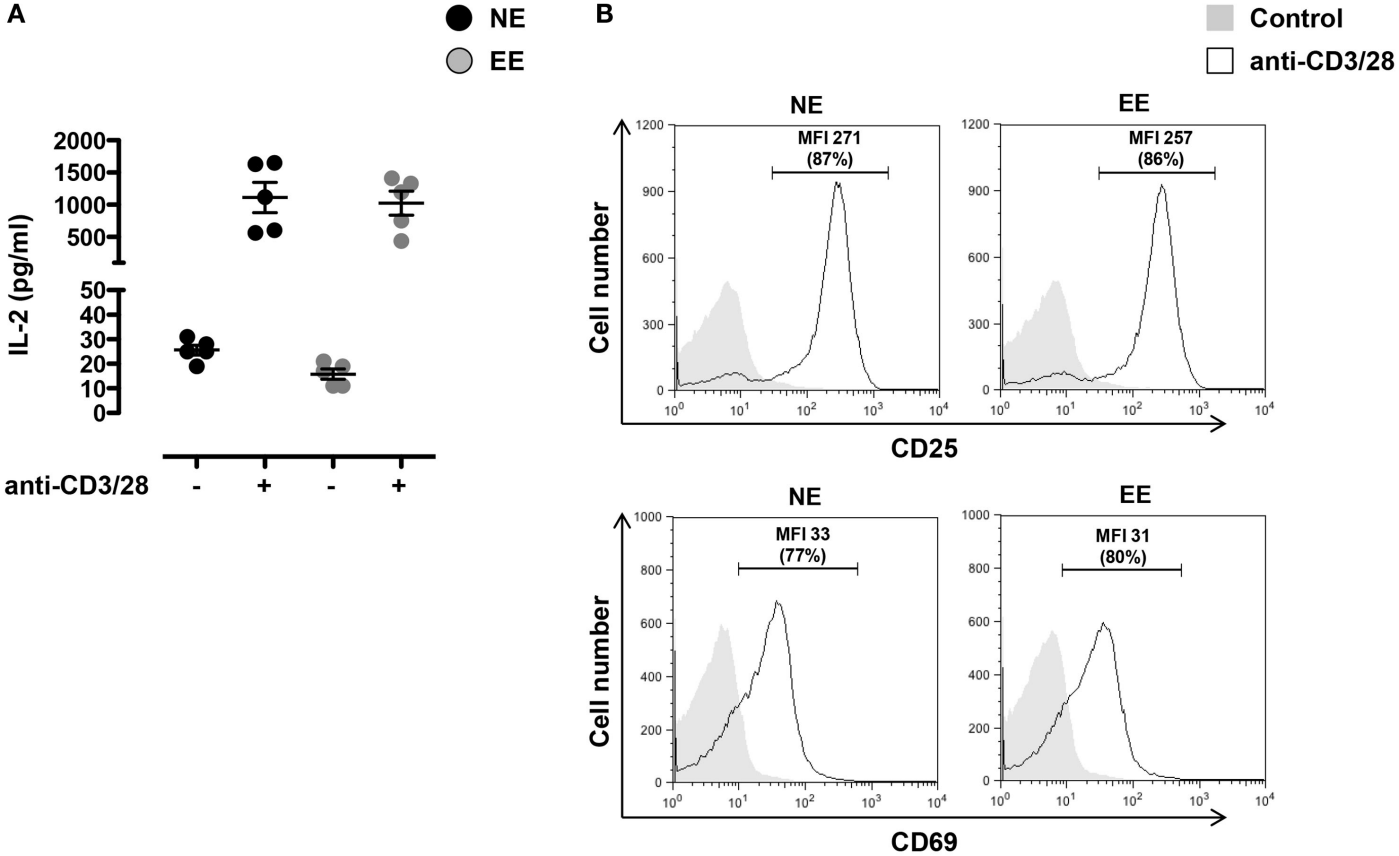
**Effect of 2 weeks EE on T cell activation *in vitro***. **(A)** Levels of IL-2 in the cell supernatants of NE and EE T cells isolated from lymph nodes stimulated with 0.5 μg/ml of plate-bound anti-CD3/CD28 for 24 h. Data shown means ± SEM from *n* = 5 mice and representative of *n* = 3 separate experiments with similar results. **(B)** NE and EE T cells were left untouched (shaded histogram) or stimulated with 0.5 μg/ml of plate-bound anti-CD3/CD28 (white histograms) for 16 h and then stained for the activation markers CD25 or CD69. The numbers in the plots show the mean fluorescence intensity (MFI) and percentage of gated cells (in brackets) of the anti-CD3/CD28 activated T cells. Data shown are from *n* = 5 mice and representative of *n* = 3 separate experiments with similar results.

To investigate the effects of EE on the early T cell activation events, we stimulated T cells from NE and EE mice with plate-bound anti-CD3 plus anti-CD28 antibodies (anti-CD3/CD28) and measured CD25 and CD69 expression on the cells and IL-2 production in the supernatant. As shown in Figure 4, no significant difference in IL-2 production across EE and NE mice groups were noted (Figure 4A). Neither the relative levels of CD25 and CD69 expression nor the percentage of CD25+ and CD69+ cells showed significant differences (Figure 4B).

**Figure 5:**
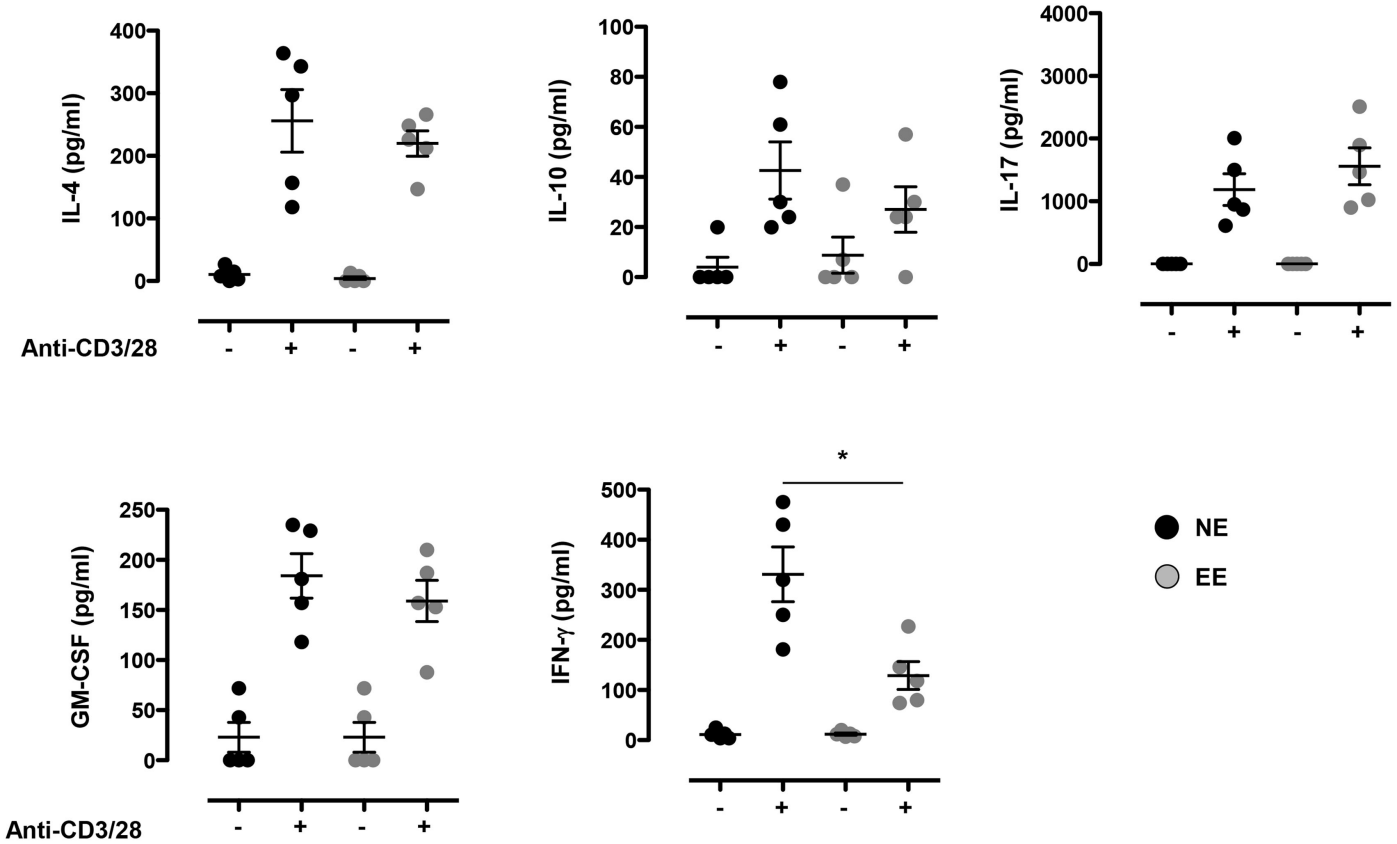
**Effect of 2 weeks EE on T cell cytokine production *in vitro***. Levels of IL-4, IL-10, IL-17, GM-CSF, and IFN-γ in the supernatants of NE and EE T cells stimulated with 0.5 μg/ml of plate-bound anti-CD3/CD28 for 24 h. Data shown means ± SEM from *n* = 5 mice and representative of *n* = 3 separate experiments with similar results. *p > 0.05.

To further explore possible differences in the activation phenotype of NE and EE T cells, we measured the levels of other classical inflammatory cytokines (IFN-γ, IL-4, IL-10, IL-17, and GM-CSF) produced after 24 h of culture. As shown in Figure 5, there were no significant differences in the levels of all the cytokines produced except for IFN-γ that was significantly lower (~2×) in EE compared to NE (*p* < 0.05; Figure 5, bottom right panel).

### Effect of Environmental Enrichment on Effector T Helper Cell Differentiation

**Figure 6:**
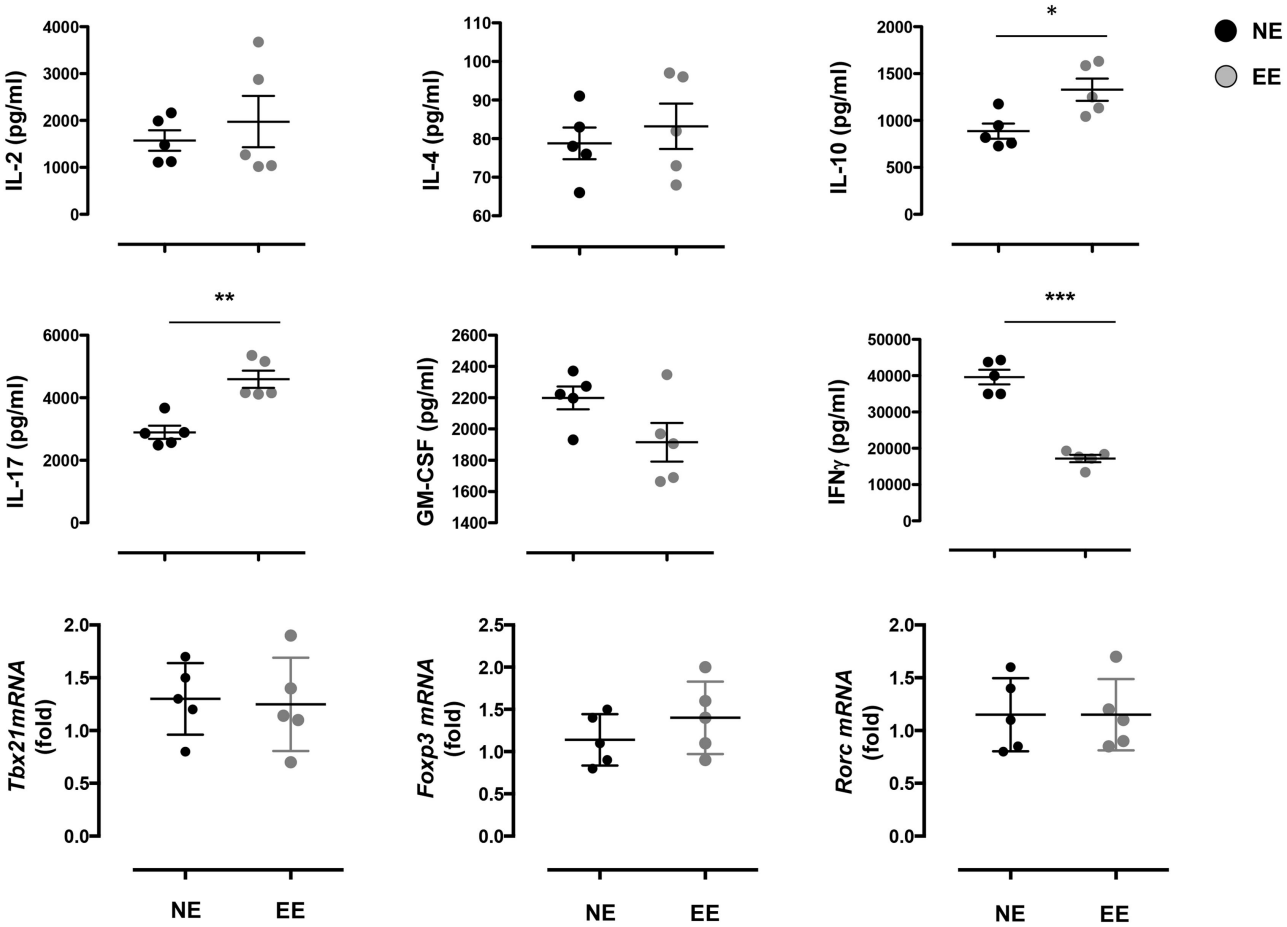
**Effect of 2 weeks EE on Th effector cell cytokine production *in vitro***. The top and the middle panels show the levels of IL-2, IL-4, IL-10, IL-17, GM-CSF, and IFN-γ in the supernatants of NE and EE T cells differentiated in Th1 skewing conditions for 4 days and then stimulated with 0.5 μg/ml of plate-bound anti-CD3/CD28 for 24 h. Data shown means ± SEM from *n* = 5 mice and representative of *n* = 3 separate experiments with similar results. *p > 0.05; **p > 0.01; ***p > 0.001. The bottom panels show the level of expression of Tbx21, Foxp3, and Rorc in the same cells. Data are expressed as fold increase of EE cells over NE cells. Values are means ± SEM from *n* = 5 mice and representative of *n* = 2 separate experiments with similar results.

**Figure 7:**
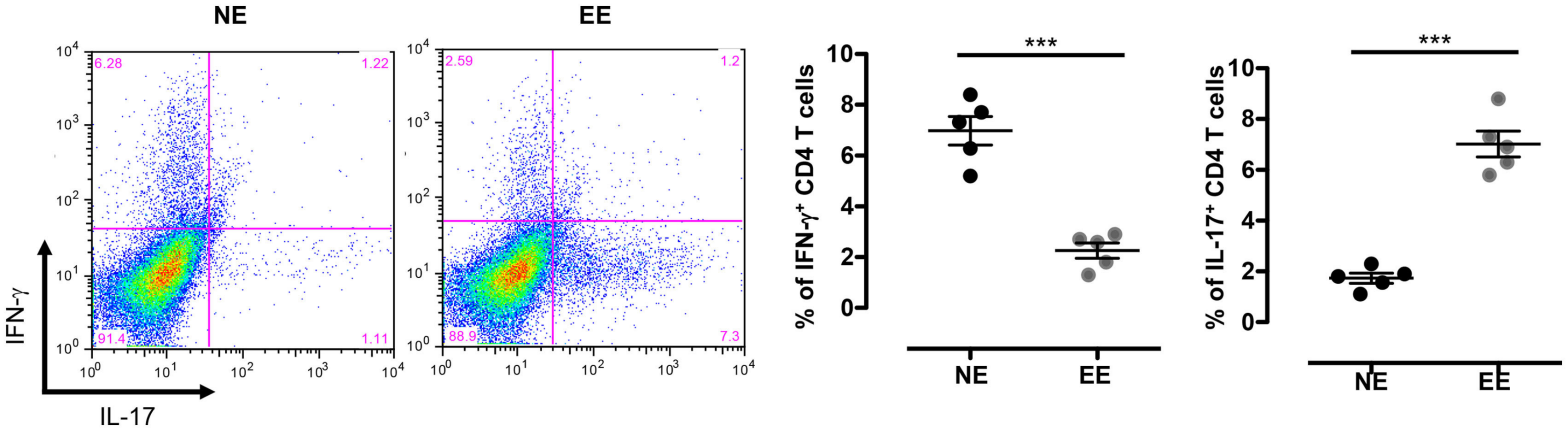
**Intracellular staining of 2 weeks EE Th effector cells obtain after differentiation in Th1 conditions *in vitro***. The dot plots on the left show gated CD4+ T cells differentiated in Th1 skewing conditions and then restimulated with ConA (5 μg/ml) for 4 h (see Materials and Methods). Cells were permeabilized and stained with antibodies against IFN-γ and IL-17. The bar graphs on right show the percentage of IFN-γ+ and IL-17+ cells. Data shown means ± SEM from *n* = 5 mice and representative of *n* = 2 separate experiments with similar results. ***p > 0.001.

The reduced production of IFN-γ in the 24-h poststimulation culture suggested the possible influence of EE on Th1 differentiation. To further test this hypothesis, we cultured NE and EE T cells for 72 h in Th1 skewing conditions. Multiplex analysis of cell supernatants of Th1 cells, restimulated overnight with anti-CD3, revealed distinct differential cytokine expression patterns between EE and NE. EE T cells released significantly lower levels of IFN-γ while producing higher levels of IL-10 and IL-17 compared to NE (Figure 6, bottom right, top right, and bottom left, respectively). FACS intracellular staining of permeabilized cells pellets confirmed these results showing an increased percentage of IL-17+ cells and decrease in IFN-γ+ (Figure 7). No significant differences were observed in cells cultured in Th2 or Th17 skewing conditions (data not shown).

### CD4 Gene Fingerprint of Enriched and Non-Enriched Mice

**Figure 8:**
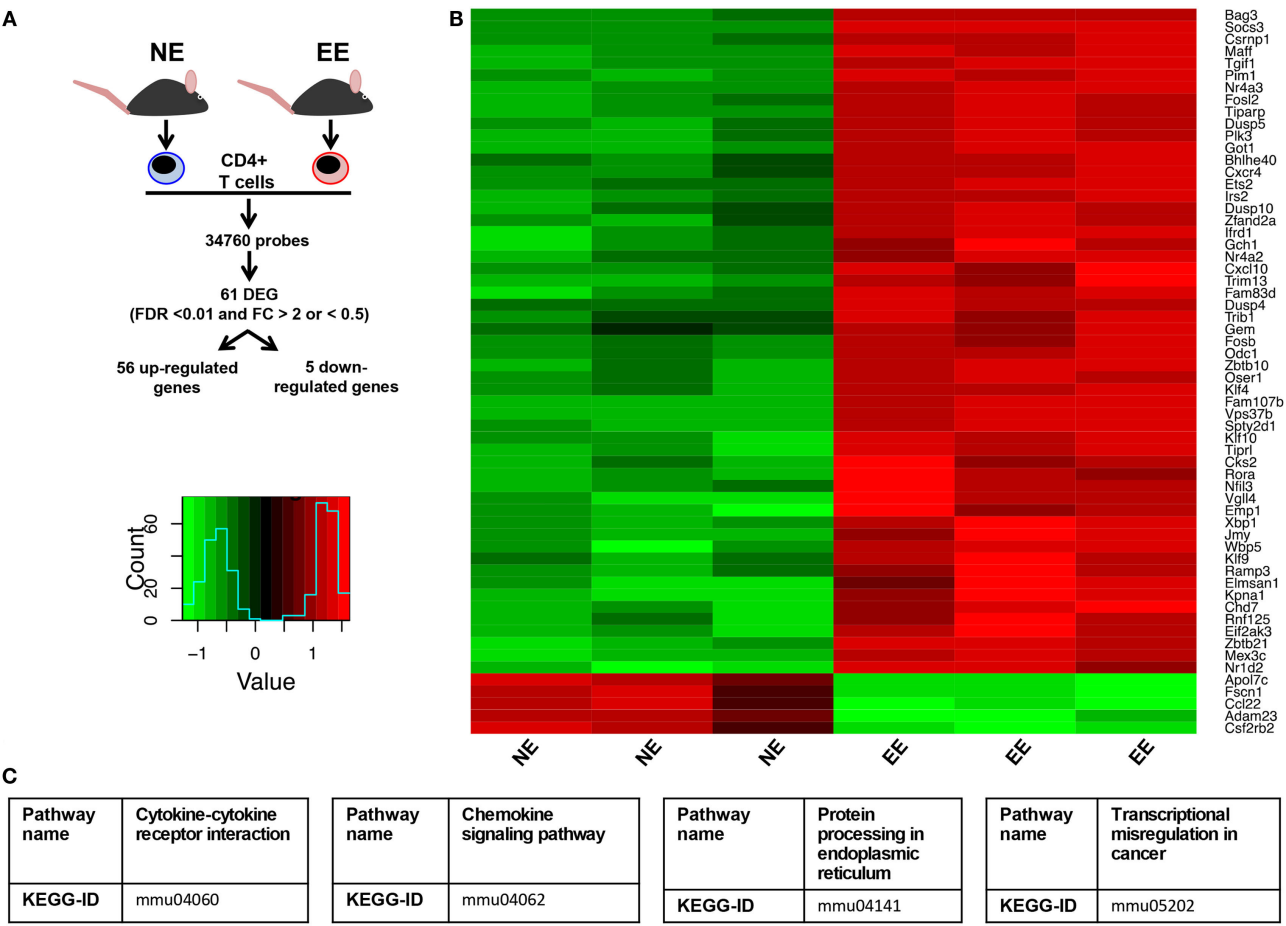
**Effect of 2 weeks EE on immunomodulatory gene expression and immune-signaling pathways**. **(A)** Schematic flowchart of the heatmap and canonical correspondence analysis on microarray data of anti-CD3–/CD28-stimulated CD4+ T cells from NE or EE mice. Genes were filtered by a moderated *t*-statistics and fold change (FC). The heatmap analysis used annotated genes only (genes with EntrezID). **(B)** Hierarchical clustering and heatmap analysis of the filtered genes EE CD4+ T cells showed a distinct cluster. **(C)** Pathway analysis identified “Cytokine–cytokine receptor interaction” (KEGG ID, mmu04060), “Chemokine signaling pathway” (KEGGID, mmu04062), “Protein processing in endoplasmic reticulum” (KEGG ID, mmu04141), transcriptional misregulation in cancer (KEGG ID, mmu05202) as a significantly altered pathway in EE animals.

**Figure 9:**
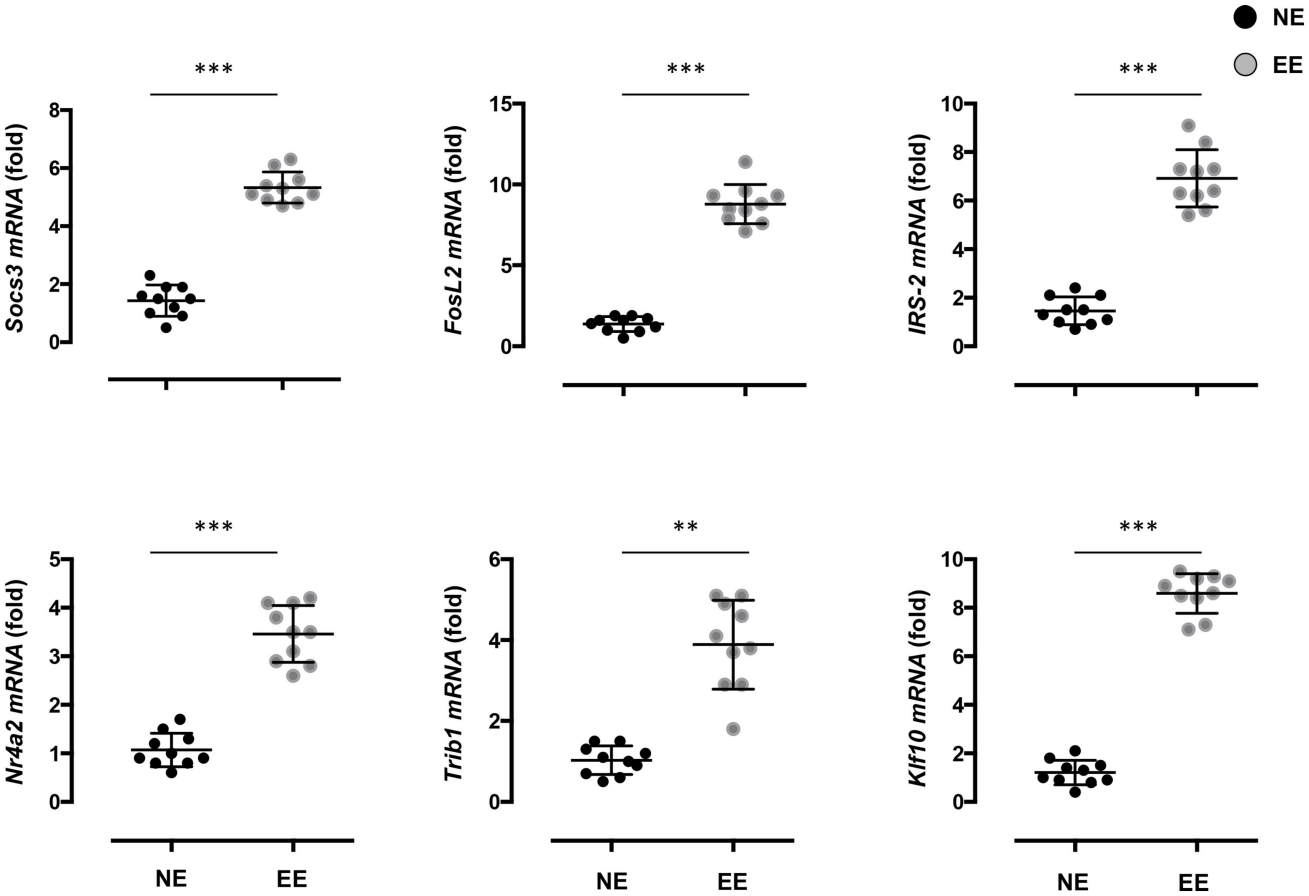
**Effect of 2 weeks EE on immunomodulatory gene expression and immune-signaling pathways**. Real-time PCR analysis of selected immunoregulatory genes of interest highlighted in the microarray. Statistical significance determined *via t*-test *p* < 0.05. Values are presented as individual data points ± SEM of 10 mice. Data are representative of two independent experiments. **p > 0.01; ***p > 0.001.

To gain further insight on the possible molecular mechanisms by which EE influenced the differentiation *ex vivo* of Th1 effector cells, we assessed the gene expression profile of anti-CD3/CD28-stimulated EE CD4+ T cells compared to their NE controls. The flowchart in Figure 8A summarizes the outcome of this analysis. From the 34,760 probes present on the chip, 61 were significantly modulated (*p* < 0.05). This corresponded to 61 DEG with a fold change (FC) value <0.5 or >2, 56 of which were upregulated and 5 downregulated. The complete list of DEG has been reported in Table S1 in Supplementary Material. RT-PCR for a selection of DEG genes (see Discussion) confirmed the results of the microarray (Figure 9).

Of these upregulated genes, a large number were identified as related to T cell activation and survival. Pathway analysis of these DEGs (listed in Table S2 in Supplementary Material) clustered them around four main categories: chemokine signaling pathways (Figure S1 in Supplementary Material), cytokine–cytokine receptor interaction (Figure S2 in Supplementary Material), protein processing in the endoplasmic reticulum (ER) (Figure S3 in Supplementary Material), and transcriptional misregulators in cancer (Figure S4 in Supplementary Material).

## Discussion

Enriched Environment is a popular tool in the field of neurobiology that has been long used to demonstrate the modulatory effect that the external environment can have on the brain and nervous system at both the functional and phenotypic level (60). In this study, we reveal that EE has similar modulatory effects in the murine immune system. More specifically, our systematic analysis of T cells – *in vivo* and *in vitro* – provides the first evidence by which EE influences the functions of these cells.

Starting from a very general comparison of the T cell immune repertoire *in vivo*, we found no readily visible or measurable difference between NE and EE mice. Indeed, looking at the T cell repertoire in both primary and secondary lymphoid organs (thymus and lymph nodes), there were no significant differences in terms of the overall number or the ratio between CD4 and CD8 cells.

Still considering any possible effect of EE *in vivo*, when we tested EE mice for possible changes in their basal state of anxiety-like behavior, we found no significant changes in corticosterone levels or in their behavioral responses in the open field or MBTs. This was surprising considering that EE has been proposed to significantly improve the health and welfare of experimental animals (61). Yet, there are a number of studies that suggest the possible changes in the emotional status of mice might not be “measurable” through classical behavioral tests or corticosterone levels (62, 63). This is especially true when the treatment applied is administered over a long period of time. One explanation for this paradox is that both behavioral responses and corticosterone levels are physiological measurements of “novel” and transitory changes of the external environment. These biological responses level off upon habituation as it has been previously reported (64).

However, our analyses of *in vitro* T cell responses provided a completely different picture. As discussed later, we performed this set of experiments to specifically rule out any “confounding” factors that could be present *in vivo* and ultimately influence T cell function. When we looked at the early TCR-driven activation events, we found no significant differences in the levels of expression of activation markers CD25 and CD69 or in the production of the proliferative cytokine IL-2. These results are consistent with other studies where no significant changes in T cell activation were observed and suggested to us that EE might not have a role in regulating these early signaling pathways (65).

Moving into exploring if EE would influence the effector phase of T cell life, we investigated differences in the levels of inflammatory cytokines (other than IL-2), in the cell supernatants of T cells stimulated with anti-CD3/CD28 for 24 or 48 h. At this stage, T cells are considered in Th0 state (66, 67) and not biased toward any of the known effector phenotypes, such as Th1, Th2, Th17, or Th-GM-CSF. Similar to a previous study, we found that EE T cells produced a significantly lower level of Th1 landmark cytokine IFN-γ, while none of the other Th-specific lead cytokines (IL-4 for Th2, IL-17 for Th17, GM-CSF for Th-GM-CSF, or IL-10 for Treg) showed any significant difference.

To further investigate this possibly specific effect of EE on Th1 skewing, we differentiated EE T cells in Th1 conditions, assessing their cytokine profile upon restimulation with anti-CD3. The results confirmed our previous observation on the reduced production of IFN-γ, this time with a much greater degree of inhibition (60% vs. 40%). Most interestingly, we observed a very unique pattern of cytokine production with EE cells releasing significantly higher levels of IL-10 and IL-17 compared to control.

Recent intriguing studies in the field of Th cell plasticity have identified a new type of Th cells that produce low level of INF-γ but higher levels of IL-17 and IL-10. These have been identified *in vivo* using IL-17A fate reporter mouse [IL-17ACRE × *Rosa26* STOPfl/fl YFP (R26YFP)] crossed with IL-17A IL-10eGFP Foxp3RFP triple reporter mouse (68). Using models of self-limiting (administration of anti-CD3 monoclonal antibody) or non-resolving (DNIL-10R transgenic mice with an impairment of IL-10R signaling in CD4 T cells) inflammation, the authors of this study have shown that these IL-10 producing Th17 lymphocytes, also known as T-regulatory 17 (Treg-17) cells, represent a specific stage of the “plastic life” of T cells. Unlike Th17 cells that are regarded as a pathogenic and pro-inflammatory (69), Treg-17 cells have been shown to play a protective and immunosuppressive function, in part through their production of IL-10 (70, 71). Based on this evidence, it is tempting to speculate that the positive and “therapeutic effects” of EE are due to their ability to favor the development of Treg-17 cells.

Aiming to gain insight on the mechanisms by which EE could favor the production of this unique pattern of cytokines *in vitro*, we decided to perform a microarray analysis of EE CD4+ T cells activated with anti-CD3/CD28. The results showed a number of interesting hits that supported the concept of a gene program favoring a Th2/T-regulatory phenotype. In addition, of the most significantly unregulated genes, a large number were identified as T cell survival factors. Notable upregulated genes included *SOCS3* a member of the suppressor of cytokine signaling family that regulates CD8+ T cell proliferation through inhibition of IL-6 and IL-27 (72). Fos-related antigen 2 (FOSL2), a transcription factor preferentially expressed during the early phase of Th2 cell proliferation, was also upregulated (73). As was insulin receptor substrate 2 (IRS-2), a cytoplasmic signaling molecule shown to play a key role in IL-4 induced T cell proliferation (74).

Several regulators of CD4+ CD25− T cell and T regulatory cell (T-Reg) differentiation were also identified as significantly upregulated in the enriched group. *Nr4a2* (Nuclear Receptor Subfamily 4, Group A, Member 2) codes NURR1 (Nuclear receptor related 1 protein), an intracellular transcription factor demonstrated to promote generation of regulatory T cell types through induction of *Foxp3*, a transcription factor commonly regarded as a master regulator of T-Reg function and development (75). The protein Tribbles 1, coded by *Trib1*, also acts upon Foxp3 co-binding with the transcription factor to enhance its T-Reg promoting function (76). In addition, Kruppel-like Transcription Factor 10, coded by *Klf10* has been shown to play an important role in the suppressor function of both CD4+ CD25− T cells and T-Regs through transactivation of the TGF-β1 and Foxp3 promoters (77).

Aiming to gain more insights out of our gene expression screening, we performed pathway analysis of EE-induced DEGs. We found striking changes in both cytokine signaling cascades and responses to chemokines. This is interesting considering that a cytokine/chemokine-driven feedback loop determines for the observed differential infiltration of Th1 and Th17 cells into inflamed tissues and organs (78). Perhaps, more surprisingly, the analysis showed activation of ER stress in EE T cells. Similar to inflammation (and tightly linked to it) ER stress has been shown to have both a positive and negative effect on the immune response. Indeed, although it is clear that severe ER stress can promote inflammation and autoimmunity, several recent studies have also shown that low levels of ER stress may actually be beneficial. ER stress promotes “*an adaptive Unfolded Protein Response (UPR) that preconditions the cell to a subsequent lethal insult: a process now known as ER hormesis*” (79). Accordingly, one might hypothesize that the action of EE is to provide a state of preconditioning that confers the host the ability to better face pathogenic or harmful challenges.

In conclusion, the key findings of this study are that a simple set of alterations to the external environment for period of time, as brief as 2 weeks, can dramatically alter the immune profile of T cells making them more prone to acquire a protective inflammatory phenotype. A current limitation of this study is that we have not investigated the occurrence of these cells *in vivo* in relevant disease conditions, such as in mouse models of autoimmune diseases, although there are already studies in the literature showing for instance therapeutic effect of EE in mouse models of multiple sclerosis (80, 81). We have purposefully not done so for two main reasons. First, we wanted to assess if the EE could induce stable changes in the gene expression profile of T cells that would be maintained *ex vivo*. Second, we wished to rule out the potentially confounding influence of the *in vivo* milieu on the fate of T cells and thus obtain a more controlled comparison between NE and EE (82). Studies employing EE T cells adoptively transferred to wild-type mice as means of cellular therapy for autoimmune disease could potentially clarify this point. Similarly, injection of bone marrow wild-type cells into an irradiated EE host would help us identify the contribution of the immune milieu on EE immunomodulatory function.

We think that this study is the first step toward a systematic analysis of the specific effects EE exerts on different immune cells isolated and taken out of their physiological context. Indeed, it remains to be determined if the effects we have seen on CD4 cell differentiation are due, at least in part, to a specific contribution of non-T cells present in the splenocyte population. Our plan is to expand these investigations and assess the effect of EE on APC-like cells, such as B cells, monocytes, and macrophages. It would be interesting to see if these cells also acquire a regulatory/immunosuppressive phenotype (e.g., B-regs, healing monocytes, M2 macrophages). The availability of these data may shed light on the possible molecular mechanisms by which positive or negative environmental conditions influence the immune response. Further, these results would likely provide a novel list of potential molecular targets that could be exploited to ameliorate the treatment of several immune inflammatory disorders.

## Author Contributions

LR, GP, and KS performed the animal studies, T cell activation, and differentiation essays. SB analyzed some of the data and contributed to the drafting of the manuscript. MO performed the microarray analysis and contributed to the drafting of the manuscript. FD designed the study, performed some *in vitro* studies, and wrote the paper.

## Conflict of Interest Statement

The authors declare that the research was conducted in the absence of any commercial or financial relationships that could be construed as a potential conflict of interest.

## Supplementary Material

Table S1 **Top ranked genes (*n* = 61) for the features of EE CD4+ T cells in comparison with NE**. The table shows the identity and statistical values of the top ranked genes that were used for heatmap analysis (Figure 8) and calculating the similarity score (EE score) for the EE CD4+ T cells phenotype compared with the NE one. Genes are ordered according to their relative contributions to the EE score (i.e., the association with EE; positive values indicate association with EE cells, while negative ones indicate association with wild type).

Table S2 **Signaling pathway impact analysis of activated EE CD4+ T cells**. Microarray data of EE and NE CD4+ T cells were analyzed by a moderate *t*-statistics and fold change and subsequently analyzed for the pathway enrichment with a bootstrap technique using the Bioconductor package, SPIA. Significantly modulated pathways were selected by a global pathway significance *p*-value with considering false discovery rate (*p* < 0.05), which combines the enrichment and perturbation *p*-values.

Figure S1 **Chemokine signaling pathway map; KEGG-ID mmu04062**. We have employed the bioconductor package SPIA that analyses both *p*-value and fold change and identifies significantly modulated pathways using Kyoto Encyclopedia of Genes and Genomes (KEGG). Red colored genes were differentially expressed between the groups (either upregulated or downregulated).

Figure S2 **Cytokine–cytokine receptor interaction map; KEGG-ID mmu04060**. We have employed the bioconductor package SPIA that analyses both *p*-value and fold change and identifies significantly modulated pathways using Kyoto Encyclopedia of Genes and Genomes (KEGG). Red colored genes were differentially expressed between the groups (either upregulated or downregulated).

Figure S3 **Protein processing in endoplasmic reticulum map; KEGG-ID mmu04141**. We have employed the bioconductor package SPIA that analyses both *p*-value and fold change and identifies significantly modulated pathways using Kyoto Encyclopedia of Genes and Genomes (KEGG). Red colored genes were differentially expressed between the groups (either upregulated or downregulated).

Figure S4 **Transcriptional misregulation in cancer map; KEGG-ID mmu05202**. We have employed the bioconductor package SPIA that analyses both *p*-value and fold change and identifies significantly modulated pathways using Kyoto Encyclopedia of Genes and Genomes (KEGG). Red colored genes were differentially expressed between the groups (either upregulated or downregulated).
