# Supplementary figures and images for: Impact of Enriched Environment on Murine T Cell Differentiation and Gene Expression Profile
Source: Front Immunol. 2016 Sep 30;7:381. doi: 10.3389/fimmu.2016.00381 (PMC5042968; doi:10.3389/fimmu.2016.00381)

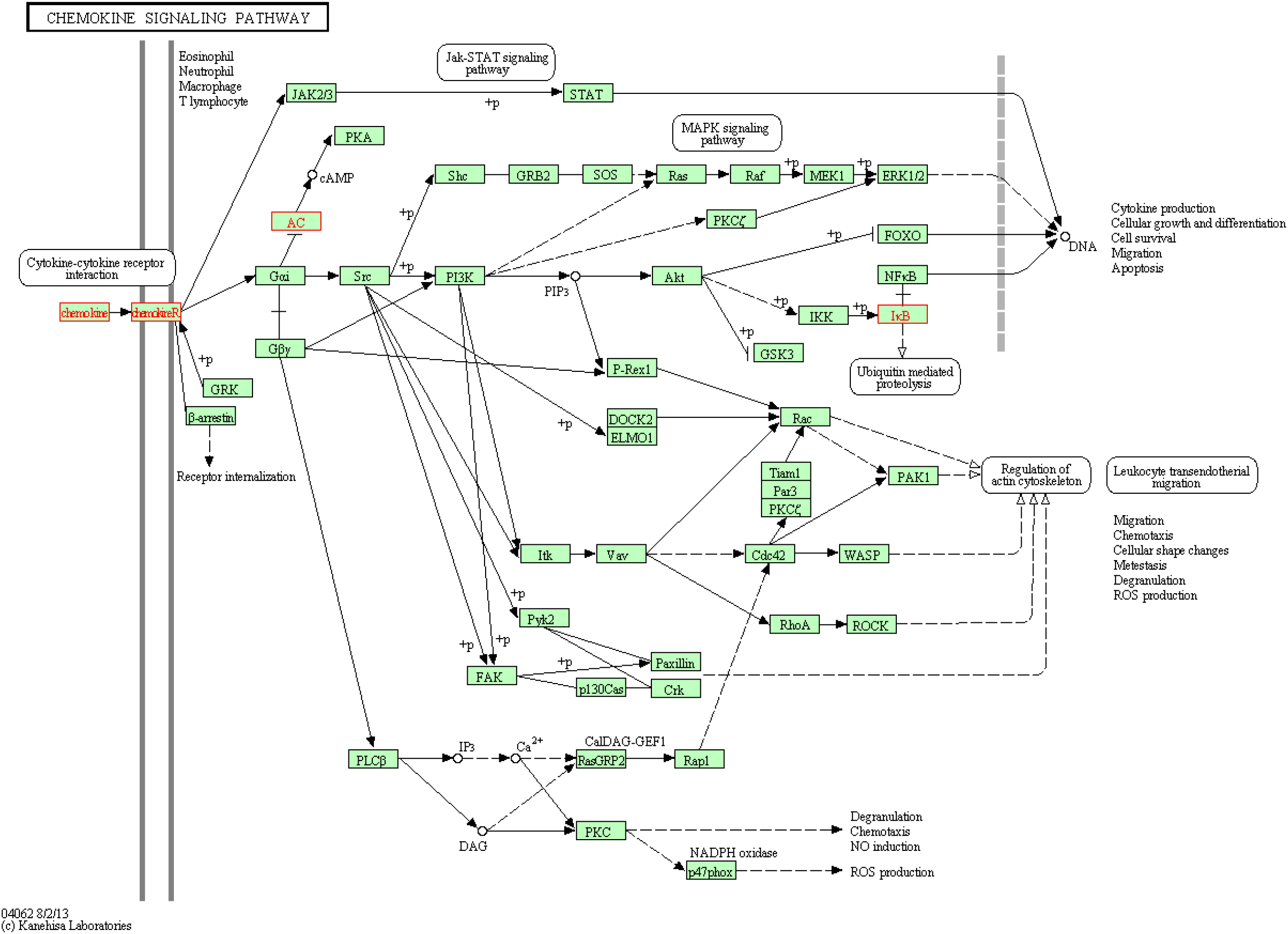

Supplement: Figure S1 — Chemokine signaling pathway map; KEGG-ID mmu04062. We have employed the bioconductor package SPIA that analyses both p-value and fold change and identifies significantly modulated pathways using Kyoto Encyclopedia of Genes and Genomes (KEGG). Red colored genes were differentially expressed between the groups (either upregulated or downregulated). [file image_1.tif]

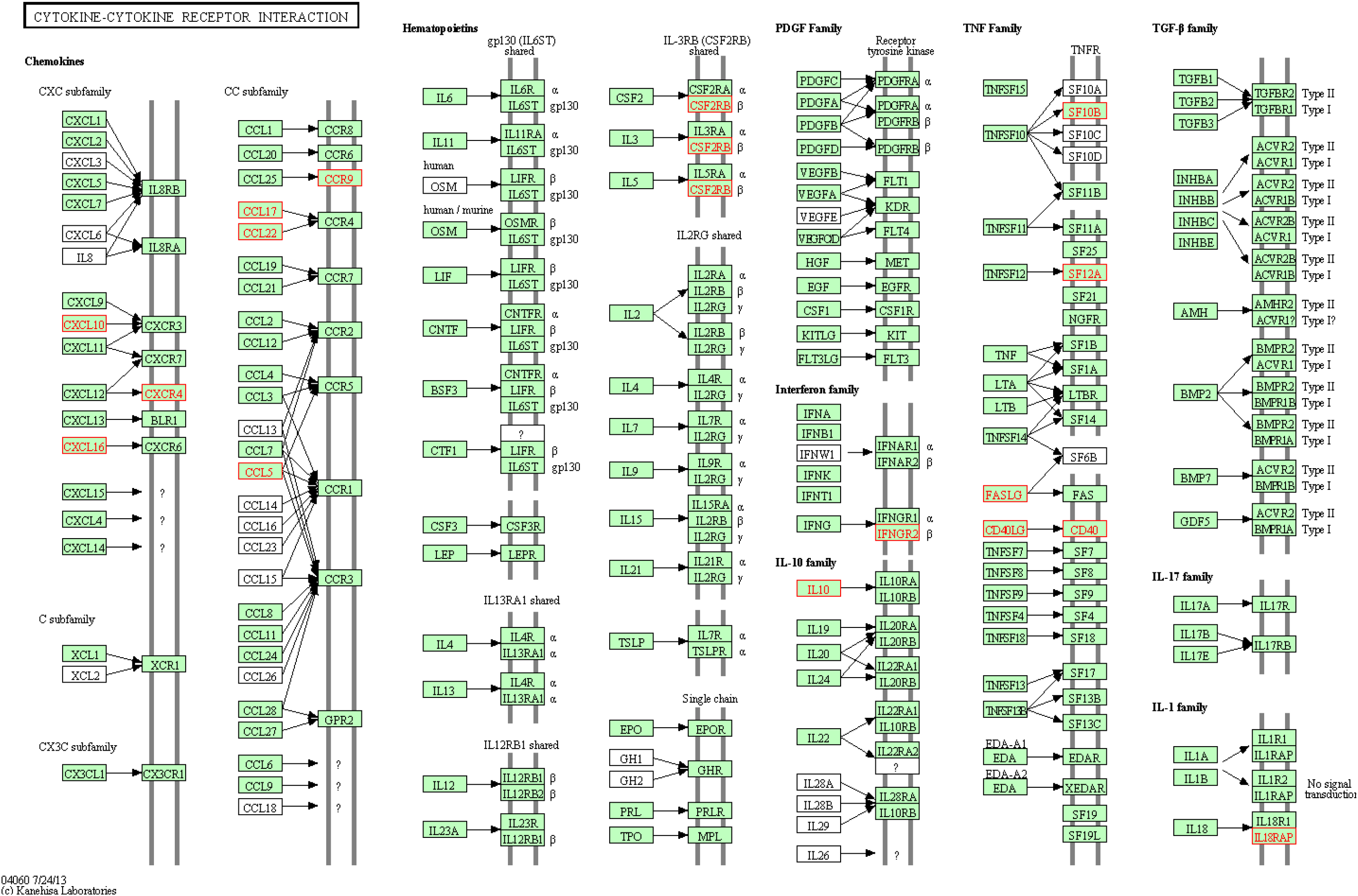

Supplement: Figure S2 — Cytokine–cytokine receptor interaction map; KEGG-ID mmu04060. We have employed the bioconductor package SPIA that analyses both p-value and fold change and identifies significantly modulated pathways using Kyoto Encyclopedia of Genes and Genomes (KEGG). Red colored genes were differentially expressed between the groups (either upregulated or downregulated). [file image_2.tif]

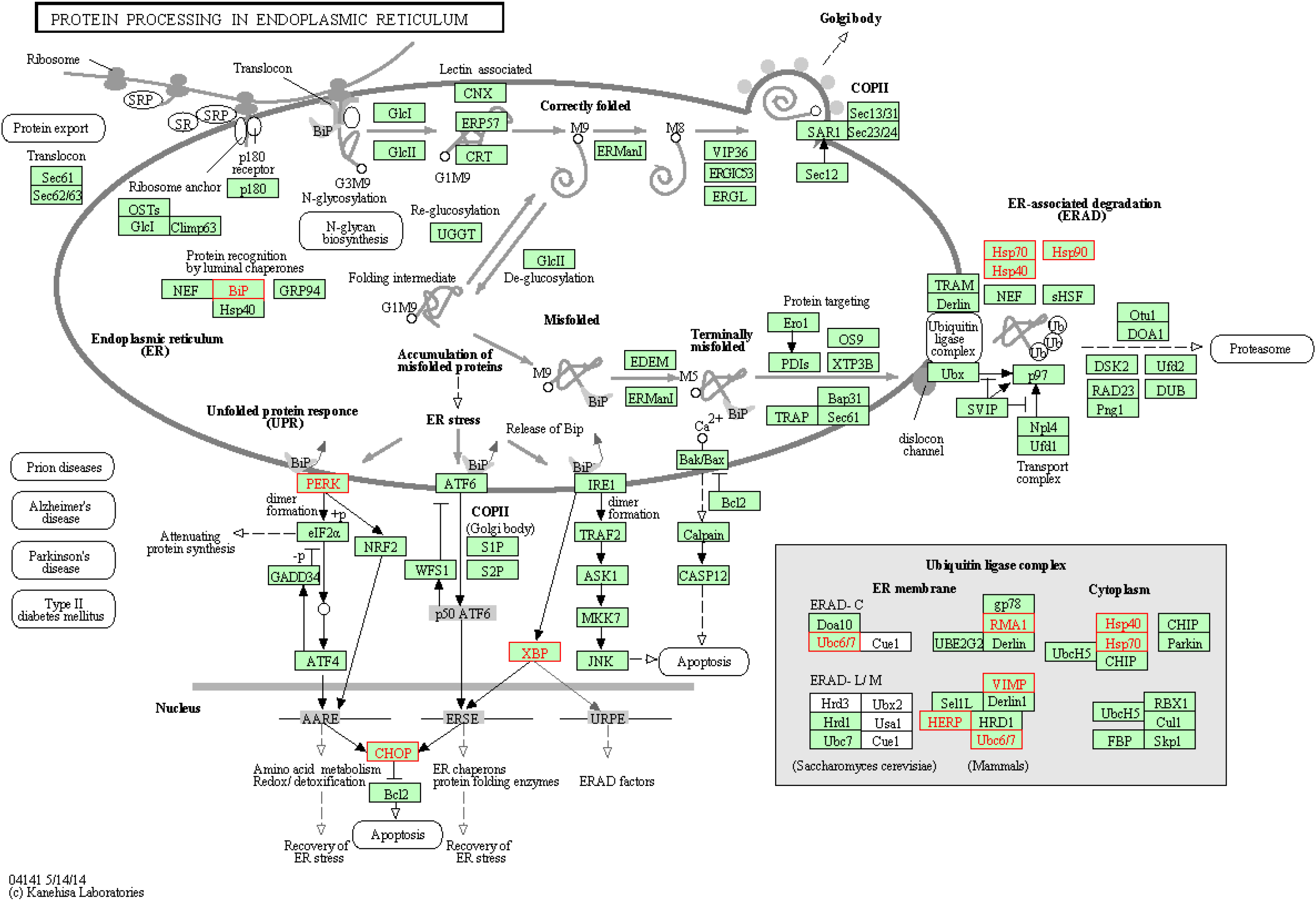

Supplement: Figure S3 — Protein processing in endoplasmic reticulum map; KEGG-ID mmu04141. We have employed the bioconductor package SPIA that analyses both p-value and fold change and identifies significantly modulated pathways using Kyoto Encyclopedia of Genes and Genomes (KEGG). Red colored genes were differentially expressed between the groups (either upregulated or downregulated). [file image_3.tif]

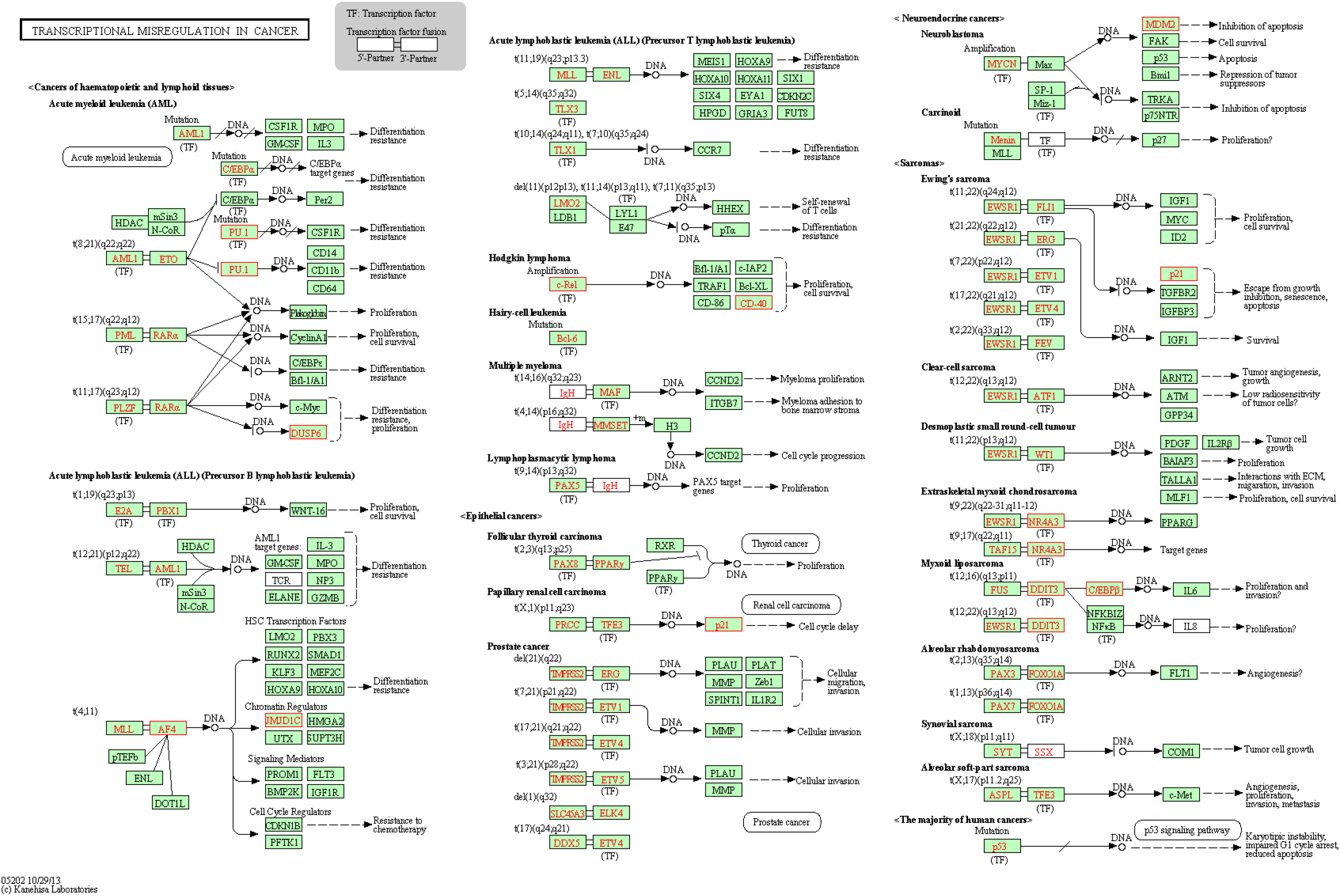

Supplement: Figure S4 — Transcriptional misregulation in cancer map; KEGG-ID mmu05202. We have employed the bioconductor package SPIA that analyses both p-value and fold change and identifies significantly modulated pathways using Kyoto Encyclopedia of Genes and Genomes (KEGG). Red colored genes were differentially expressed between the groups (either upregulated or downregulated). [file image_4.tif]
